# Associations between diagnostic time intervals and health-related quality of life, clinical anxiety and depression in adolescents and young adults with cancer: cross-sectional analysis of the BRIGHTLIGHT cohort

**DOI:** 10.1038/s41416-022-01698-6

**Published:** 2022-02-22

**Authors:** Alice S. Forster, Annie Herbert, Minjoung Monica Koo, Rachel M. Taylor, Faith Gibson, Jeremy S. Whelan, Georgios Lyratzopoulos, Lorna A. Fern

**Affiliations:** 1 Epidemiology of Cancer Healthcare and Outcomes (ECHO) Group, Department of Behavioural Science and Health, University College London, London, WC1E 6BT UK; 2 Medical Research Council, Integrative Epidemiology Unit at University of Bristol, Oakfield House, Oakfield Grove, Bristol, BS8 2BN UK; 3 Population Health Sciences, University of Bristol, Bristol, BS8 2BN UK; 4 Centre for Nurse, Midwife and Allied Health Profession Led Research (CNMAR), University College London Hospitals NHS Foundation Trust, London, UK; 5 School of Health Sciences, Faculty of Health and Medical Sciences, University of Surrey, Guildford, Surrey GU2 7YH UK; 6 Centre for Outcomes and Experience Research in Children’s Health, Illness and Disability (ORCHID), Great Ormond Street Hospital for Children NHS Foundation Trust, London, WC1N 3JH UK; 7 Cancer Clinical Trials Unit, University College London Hospitals NHS Foundation Trust, London, UK

**Keywords:** Diagnosis, Cancer, Cancer epidemiology

## Abstract

**Background:**

The association of diagnostic intervals and outcomes is poorly understood in adolescents and young adults with cancer (AYA). We investigated associations between diagnostic intervals and health-related quality of life (HRQoL), anxiety and depression in a large AYA cohort.

**Methods:**

Participants aged 12–24 completed interviews post-diagnosis, providing data on diagnostic experiences and the patient-reported outcomes (PROs) HRQoL, anxiety and depression. Demographic and cancer information were obtained from clinical and national records. Six diagnostic intervals were considered. Relationships between intervals and PROs were examined using regression models.

**Results:**

Eight hundred and thirty participants completed interviews. In adjusted models, across 28 of 30 associations, longer intervals were associated with poorer PROs. Patient intervals (symptom onset to first seeing a GP) of ≥1 month were associated with greater depression (adjusted odds ratio (aOR):1.7, 95% Confidence Interval (CI):1.1–2.5) compared to <1 month. ≥3 pre-referral GP consultations were associated with greater anxiety (aOR:1.6, CI:1.1–2.3) compared to 1–2 consultations. Symptom onset to first oncology appointment intervals of ≥2 months was associated with impaired HRQoL (aOR:1.8, CI:1.2–2.5) compared to <2 months.

**Conclusions:**

Prolonged diagnostic intervals in AYA are associated with an increased risk of impaired HRQoL, anxiety and depression. Identifying and delivering interventions for this high-risk group is a priority.

## Background

Adolescents and young adults (AYA) with cancer are a unique group warranting specialist care and attention [1]. The lower age of AYA is generally accepted to be around 12/13 years, but the upper range of young adulthood ranges from 24 up to 39 years depending on jurisdiction [1]. While cancer remains relatively rare among AYAs, the incidence is increasing worldwide [2, 3]. Five-year survival for AYA ranges from 50 to 98% in high-income countries depending on cancer type, but improvements in outcomes over the last 20 years have been modest for most solid cancers and lag behind improvements observed in children and some older adult cancers [4]. Deficits in outcome improvements are thought to be associated with cancer biology, prolonged diagnostic pathways/intervals, lack of access to research and place of care [5–8]. When treatment is successful, societal gains are potentially long-lasting and economically beneficial given life years gained. However, treatment and disease-related morbidity are considerable, as are interruptions to social and psychological development, education and employment, highlighting the importance of considering patient-reported outcomes (PROs) such as health-related quality of life (HRQoL), anxiety and depression alongside clinical outcomes, such as survival [7, 9–12].

A timely cancer diagnosis is pivotal to international cancer control strategies, the premise being that shorter diagnostic intervals lead to improved patient outcomes including survival and HRQoL [13]. Adolescents and young adults diagnosed with cancer often experience prolonged convoluted diagnostic pathways compared with children and older adults [6, 15–17]. Consequently, improving the diagnostic experience is listed within the Top 5 priority research questions identified by AYA, carers and professionals in the United Kingdom where AYA are defined as those aged 13–24 years at diagnosis [18]. Despite this, limited evidence exists regarding outcomes that can be improved by early diagnosis interventions for AYA, and the magnitude of outcome gains such interventions may deliver [16, 19, 20]. Most studies lack appropriate theoretical framing and have poorly defined time intervals [16].

Associations between prolonged diagnostic intervals and poorer clinical and psychological outcomes have been described for adults [14], and multiple General Practitioner (GP) consultations are associated with poorer experiences of care [21]. Young people are a unique population with distinct disease and psychological features [1, 8]. Teenage years and early adulthood are associated with concentrated challenges in social, emotional and educational development, and stresses generated by entry to the job market, and independent living [22–24]. While a cancer diagnosis is a major life event at any age, we hypothesise it can be particularly disruptive to young people’s lives. Lack of cancer knowledge, and a developing sense of embodied self and identity are at an early stage of maturity [25]. For these reasons, it cannot be inferred that outcomes associated with diagnostic intervals in older adults will be similar in AYA therefore empirically examining the psychological impact of late diagnosis in AYA is justified due to increasing psychological maturity and different cancer types experienced by AYA. The aim of this study was to investigate diagnostic intervals and their association with HRQoL, anxiety and depression in a large well-characterised cohort of AYA aged 13–24 years at diagnosis [9].

## Methods

### Study design and participants

We conducted a cross-sectional secondary analysis of the national BRIGHTLIGHT AYA cohort data. The cohort profile and detailed recruitment methods have been reported previously [7, 9]. BRIGHTLIGHT was a National Institute for Health Research-funded programme of research (RP-PG-1209-10013) undertaken to determine whether specialist care for AYA with cancer was associated with improved outcomes [7, 9].

BRIGHTLIGHT recruited AYA, diagnosed in England and aged 13–24 at the time of any new cancer diagnosis (International Classification of Diseases, 10th Revision codes C00–C97) [9]. Participants diagnosed between July 2012-December 2014 were recruited from 97 hospitals, some of which were specialist AYA centres. Young people were ineligible to participate if they were unable to complete the survey, could not give consent, if they were anticipated to die within six months of diagnosis or serving a custodial sentence [9].

Ethical approval was granted by the London Bloomsbury Research Ethics Committee (11/LO/1718). Adolescents and young adults gave written consent, while parental consent was obtained for those <16 years to participate in the survey and for clinical information from their medical records and NHS databases to be collected. Approval was given for additional data to be obtained from the Office for Data Release by the Confidentiality Advisory Group with a further amendment to enable secondary data analysis of anonymised data (reference ECC 8-05(d)/2011).

### Procedures

BRIGHTLIGHT cohort data were collected from multiple sources: a bespoke patient survey, case report forms (CRF), and data from the National Cancer Registration and Analysis Service (NCRAS) in Public Health England (PHE).

The BRIGHTLIGHT survey was designed to capture young people’s experiences of cancer care and consisted of 15 domains identified by AYA as important, including five validated questionnaires and questions about their experience before and during diagnosis [26]. Survey data were collected at five time points (waves) over 3 years, the first survey was administered by face-to-face interviews carried out by a researcher from an independent research company. The subsequent surveys were administered by telephone or online. We used diagnostic experience and patient-reported outcome data from the first time point, which was 5–7 months after diagnosis collected during the face-to-face interviews. The BRIGHTLIGHT survey is available under licence from https://xip.uclb.com/i/healthcare_tools/brightlight_wave1.html.

Clinical teams completed CRFs reflecting care delivered in the first 12 months after diagnosis. This also included gender, age at diagnosis, home postcode (matched to Local Super Output Area and used to derive Index of Multiple Deprivation (IMD) 2015 scores, a measure of socioeconomic status [27]), and self-reported ethnicity. Electronic health record data were extracted from NCRAS, which was used to validate, and supplement data completed in the CRFs and included date of diagnosis, tumour type, morphology, staging and treatment.

### Principal exposure variables: measures and markers of diagnostic timeliness

**Fig. 1:**
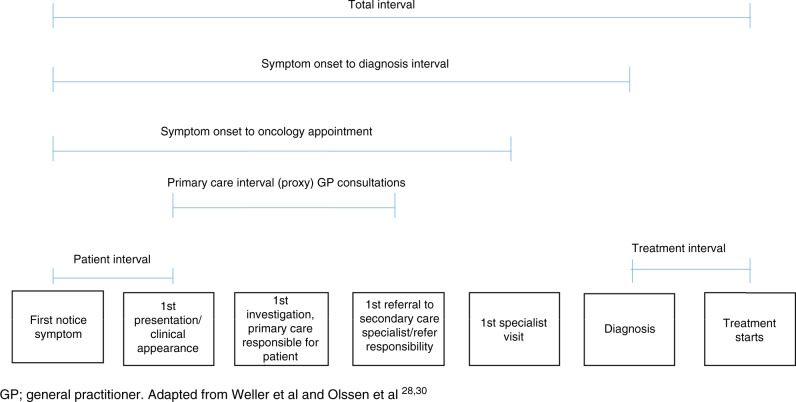
Exposure definitions and their relation to the diagnostic/treatment pathway.

We used six survey questions, date of diagnosis from the NCRAS population-based cancer registry and the start of treatment date from the CRF to inform six interval measures based on recognised diagnostic and treatment intervals defined by previous international consensus and our previous work on AYA diagnostic intervals; [6, 28–31] (Table 1; Fig. 1). In addition to diagnostic intervals, we included measures of both treatment interval and total interval. Although these are likely to be correlated, we felt it important to assess the treatment interval to allow comparison across studies and the total interval as this is a current policy focus [32, 33].

**Table 1:** Exposure variables (intervals), definitions, data source measurement, categories and rationale for how they were treated.

Interval	Definition	Data source	Measurement	Categories included in analysis	Rationale for how treated in analysis	Total n in analysis
Patient interval	Time from patient noticing a symptom to seeking help	Patient survey^a^	(1) Under 1 week (2) 1 week up to 2 weeks (3) Over 2 weeks up to 4 weeks (4) Over 1 month up to 3 months (5) Over 3 months up to 6 months (6) Over 6 months up to 12 months (7) More than 12 months (8) Don’t know/can’t remember	<1 month v. ≥1 month	We chose a 1 month cut off, deeming this long enough to be clinically important, and taking into consideration that public health education campaigns about awareness of cancer symptoms typically use a cut off of 3 weeks or longer for the duration of a new symptom as a prompt to seek help [6]	719
Proxy measure of primary care interval [26]	Number of GP consultations, after first seeking help about symptoms and before referral to secondary care	Patient survey^a^	Number of GP consultations	1–2 v. ≥3	Three or more pre-referral consultations are reported to be associated with poorer experiences of care [17]. The number of GP consultations and primary care interval are correlated [31]	676
Symptom onset to oncology interval	Time from first noticing a symptom to a first oncology appointment	Patient survey^a^	(1) Less than 1 month (2) Over 1 up to 2 months (3) Over 2 up to 3 months (4) Over 3 up to 6 months (5) More than 6 months (6) Don’t know/Can’t remember	<2 months v. ≥2 months	We used the threshold of 2 months by summing and rounding up: -The most commonly reported number of weeks participants in BRIGHTLIGHT waited from noticing a symptom to being seen in primary care was 2- weeks, 48% [6] -The median primary care interval in adults with 2 consultations is 15 days [31], the median number of GP consultations in the BRIGHTLIGHT cohort is 2. -The nationally mandated NHS target for patients to be seen by a specialist following referral for suspected cancer is 2 weeks [33]	794
Symptom onset to diagnosis interval	Time from first noticing a symptom to receiving a diagnosis	Patient survey^a^, PHE data	Days	Short 0–4 weeks Medium 5–11 weeks Long ≥12 weeks	Based on previously published adult literature classifying the diagnostic interval (presentation to diagnosis) as short, medium and long [29]	770
Total interval	Time from first noticing a symptom to starting treatment	Patient survey^a^, CRF	Days	<91 days v. ≥91 days	We used the threshold of 91 days by summing: -The most commonly reported number of weeks participants in BRIGHTLIGHT waited from noticing a symptom to being seen in primary care (2 weeks, 48%) -The median primary care interval in adults with 2 consultations is 15 days [31], the median number of GP consultations in BRIGHTLIGHT -The nationally mandated target from urgent referral to treatment in England (62 days) [33]	577
Treatment interval	Time from diagnosis to starting treatment	PHE, CRF	Days	≤31 days v. >31 days Reported in supplementary material only (Table S3)	The nationally mandated target from the decision to treat to treatment starting in England (31 days) [33]	550

^a^Administered through face-to-face interview.

*CRF* case report form, *GP* General Practitioner, *PHE* Public Health England.

### Patient-reported outcomes: health-related quality of life, anxiety and depression

The primary outcome, HRQoL, was defined according to a previous conceptual definition and was measured using Pediatric Quality of Life Questionnaire (PedsQL), which was the only validated measure for HRQoL for ages 13–24 at study onset [34–36]. The PedsQL consists of 23 items rated using a 5-point Likert Scale (never, almost never, sometimes, often and almost always). Responses can be presented as four domain scores (physical, emotional, social and work/studies functioning), two summary scores (physical and psychosocial function) and a total score. Domain, summary and total scores range from 0 to 100, with 100 representing the best possible HRQoL [7, 9, 35, 36]. We used total scores to categorise participants into two groups, using pre-defined cut-offs of >69.7 for low and <69.7 for high risk of impaired HRQoL respectively [9, 35, 36].

Anxiety and depression were assessed using the Hospital Anxiety and Depression Scale (HADS) [37, 38]. This consists of seven items for anxiety and seven items for depression, scoring for each item ranging from zero to three, with three denoting the highest anxiety or depression levels (maximum score of 21 on the anxiety and depression subscales, respectively). Based on published literature we categorised participants as meeting clinical thresholds (total subscale score <8 versus ≥8) and treatment thresholds (total subscale score <11 versus. ≥11) [31].

### Potential confounding variables

Additional variables adjusted for in the analysis were: gender (male/female); age (12–15 years, 16–18 years, 19–24 years); ethnicity (White v not White); Index of Multiple Deprivation (IMD) quintile; relationship status (married, civil partnership or cohabiting; single or divorced); employment status (education; working full- or part-time); other work (apprentice, internship or voluntary); not seeking work; unemployed; long-term sick; cancer site/type (grouped according to Birch’s morphology-based classification for AYA cancers, which more accurately reflects the cancer types for those aged 13–24 [32]) and treatment type (systemic anti-cancer therapy (SACT) only; SACT and surgery; SACT and radiotherapy; SACT and radiotherapy and surgery; surgery only; surgery and radiotherapy; radiotherapy only; transplant and ‘other’). We also adjusted for the amount of inpatient care received in an AYA specialist centre using NHS Hospital Episodes Statistics admitted patient care data as previously described [7, 9]. Specifically, the amount of AYA specialist care was categorised as ‘all’ (all admitted care delivered in an AYA specialist centre); ‘no’ (no admitted care in an AYA specialist centre), or ‘some’ (some admitted care delivered in an AYA specialist centre with additional care in a children’s or adult cancer centre).

### Analysis

Participants for whom interval information was missing were excluded from that specific interval analysis (see Table 1 for total included participants for each interval variable). We use confidence intervals (CI) to determine the precision of our estimates post-analysis, given our fixed sample size.

We describe the distribution of the sample in terms of patient-reported outcomes and interval data reporting numerators with frequencies or means with standard deviation (SD).

To explore if there are differences in patient-reported outcomes by diagnostic intervals, we fitted crude and adjusted logistic regression models, reporting odds ratios with 95% CI. Adjusted models included gender, age group, deprivation, ethnicity, cancer site/type, marital status and employment status. For HRQoL, adjustment was also made for treatment type and category of AYA specialist care, as the latter was significantly associated with HRQoL in the primary BRIGHTLIGHT analysis [7].

All analyses were conducted in STATA version 15 [39].

### Patient and public involvement

Two online workshops with eight members of the BRIGHTLIGHT Young Advisory Panel (YAP, report in preparation) were held. One at the beginning of the project to guide analysis and again at the end to aid interpretation of results.

### Role of funding source

Study funders had no role in study design, data collection, data analysis, data interpretation or manuscript writing. The corresponding author had full access to all the data in the study and had final responsibility for the decision to submit for publication.

## Results

A total of 1114 AYA aged 12–24 consented to participate in BRIGHTLIGHT of whom 830 completed the wave 1 survey (75%) [9]. Reasons for dropout between consent and interview included early death, refusal and illness; these participants were not atypical of those who remained in the study [6]. Five patients were recruited aged 12, due to discrepancies in dates of diagnosis between the recruiting centre and NCRAS data but were included as they were close to their 13th birthday.

### Sample description

As previously described [7, 9] the BRIGHTLIGHT cohort included information on the diagnostic routes/intervals and the symptom profiles for the 830 participants who completed wave 1 [6, 40]. The mean patient age was 19.6 years (SD 3.27), 453 (55%) were male and the majority were from a White ethnic background (730, 88%). Lymphoma was the most common cancer type (*n* = 266; 32%), followed by germ cell tumours (*n* = 156; 19%) and leukaemia (*n* = 105; 13%). Supplementary file, Table S1, details demographic characteristics and a summary of variables adjusted for.

Most participants saw their GP within 1 month of noticing a symptom (patient interval, *n* = 533; 73%) and over a third had ≥3 GP consultations prior to referral (*n* = 242; 35%). Nearly half (47%) waited longer than 2 months from first noticing a symptom to first oncology appointment (‘symptom onset to oncology’). The median time from first noticing a symptom to diagnosis (‘symptom onset to diagnosis’) was 62 days (IQR: 29–152 days), with 44% having a ‘symptom onset to diagnosis’ interval of 12 or more weeks. The median total interval (symptom onset to start of treatment) was 95 days (IQR: 41–196 days) and the majority, 59%, had a treatment interval ≥91 days. See Table 2 for a full description of the outcomes and exposures in the sample.

**Table 2:** Description of the outcomes and exposures in the sample.

	*n* (%)
*Exposures*^a^	
Patient interval	
<1 month	544 (73)
≥1 months	204 (27)
GP consultations (number)	
1–2	459 (65)
3+	242 (35)
Symptom onset to oncology appointment	
<2 months	439 (53)
≥2 months	388 (47)
Symptom onset to diagnosis interval	
Short (0–4 wks.)	197 (25)
Medium (5–11 wks.)	251 (31)
Long (≥12 wks.)	355 (44)
Symptom onset to treatment interval	
<91 days	248 (41)
≥91 days	353 (59)
Outcomes	
HRQuality of life PedsQL,	
Total sample (mean 66.20, sd 19.79)	829 (100)
Low risk of impaired HRquality of life (>69.7)	366 (44)
High risk of impaired HRquality of life (<69.7)	463 (56)
Depression HADS	
Total sample (mean 4.62, sd 3.68)	829 (100)
Not clinically depressed (<8)	655 (79)
Clinically depressed (>8)	175 (21)
Anxiety HADS	
Total sample (mean 6.89, sd 4.39)	830 (100)
Not clinically anxious (<8)	498 (60)
Clinically anxious (>8)	332 (40)

*PedsQL* Pediatric Quality of Life Questionnaire, *HR* health-related, *HADS* Hospital Anxiety and Depression Scale, *GP* General Practitioner, *sd* standard deviation.

^**a**^Summed numerators are less than the denominator due to missing data.

### Diagnostic and treatment intervals and patient-reported outcomes

**Fig. 2:**
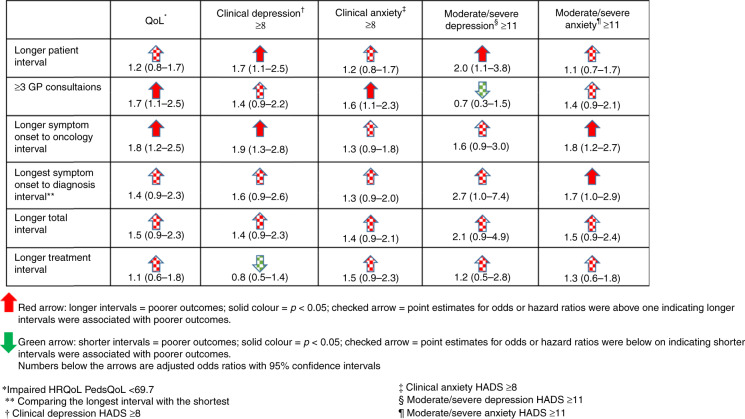
Overview of the relationship between diagnostic and treatment timeliness patient-reported outcomes. GP general practitioner.

Figure 2 depicts the relationships between diagnostic/treatment intervals and patient-reported outcomes.

### Diagnostic intervals and HRQoL (Table 3)

**Table 3:** Crude and adjusted logistic regression models exploring associations between HRQoL and diagnostic and treatment intervals.

	High risk of impaired HRQoL *n* (%)	Low risk of impaired HRQoL *n* (%)	Crude OR (95% CI)	Adjusted^a^ OR (95% CI)	*p*-value (adjusted value)
Patient interval					
<4 weeks	299 (55)	245 (45)	1 (Ref)	1 (Ref)	
≥4 weeks	188 (58)	86 (42)	1.1 (0.8–1.6)	1.2 (0.8–1.7)	0.48
GP consultations (number)					
1–2	218 (48)	241 (53)	1 (Ref)	1 (Ref)	
3+	164 (68)	77 (32)	2.4 (1.7–3.3)	1.7 (1.1–2.5)	0.01
Symptom onset to oncology					
<2 months	210 (48)	228 (52)	1 (Ref)	1 (Ref)	
≥2 months	252 (65)	136 (35)	2.0 (1.5–2.7)	1.8 (1.2–2.5)	<0.01
Symptom onset to diagnosis interval					
Short (0–4 wks.)	103 (53)	93 (47)	1 (Ref)	1 (Ref)	
Medium (5–11 wks.)	118 (47)	133 (53)	0.8 (0.6–1.2)	0.8 (0.5–1.3)	0.37
Long (≥12 wks.)	229 (54)	126 (35)	1.6 (1.2–2.3)	1.4 (0.9–2.3)	0.12
Total interval					
<91 days	152 (53)	137 (47)	1 (Ref)	1 (Ref)	
≥91 days	198 (64)	113 (36)	1.6 (1.1–2.2)	1.5 (0.9–2.3)	0.09

*HRQoL* health-related quality of life, *CI* confidence interval.

^a^Adjusted for gender, age group, deprivation, ethnicity, cancer site/type, marital status, education, treatment type and category of AYA specialist care.

Participants with ≥3 GP consultations prior to referral and those with a ‘symptom onset to oncology’ interval of ≥2 months were more likely to be at high risk of impaired HRQoL in crude and adjusted models (adjusted odds ratio (aOR) 1.7, 95% CI: 1.1–2.5, aOR: 1.8, 95% CI: 1.2–2.5, respectively). The point estimates of aORs were consistently above one (higher risk of low HRQoL) for those with a longer patient interval, ‘symptom onset to diagnosis’ interval and the total interval, although there was insufficient evidence of group differences. Findings for the treatment interval are shown in Table S2.

### Diagnostic intervals and depression (Table 4)

**Table 4:** Crude and adjusted logistic regression models exploring associations between diagnostic and treatment intervals and depression and anxiety.

	Depression					Anxiety				
	Not clinically depressed Mean: 3 SD: 2.2 *n* (%)	Clinically depressed Mean: 10 SD: 2.4 *n* (%)	Crude OR (95% CI)	Adjusted^a^ OR (95% CI)	Adjusted *p*-value	Not clinically anxious Mean: 4 SD: 2.2 *n* (%)	Clinically anxious Mean: 11 SD: 2.9 *n* (%)	Crude OR (95% CI)	Adjusted^a^ OR (95% CI)	Adjusted *p*-value
Patient interval										
<4 weeks	442 (81)	102 (19)	1 (Ref)	1 (Ref)		331 (61)	213 (39)	1 (Ref)	1 (Ref)	
≥4 weeks	148 (73)	56 (27)	1.6 (1.1–2.4)	1.7 (1.1–2.5)	0.02	111 (54)	93 (46)	1.3 (0.9–1.8)	1.2 (0.8–1.7)	0.40
GP consultations (number)										
1–2	378 (82)	81 (18)	1 (Ref)	1 (Ref)		301 (66)	158 (34)	1 (Ref)	1 (Ref)	
≥3	181 (75)	61 (25)	1.6 (1.1–2.3)	1.4 (0.9–2.2)	0.11	126 (52)	116 (48)	1.8 (1.3–2.4)	1.6 (1.1–2.3)	0.01
Symptom onset to oncology										
<2 months	371 (85)	68 (15)	1 (Ref)	1 (Ref)		282 (64)	157 (36)	1 (Ref)	1 (Ref)	
≥2 months	282 (73)	106 (27)	2.1 (1.5–2.9)	1.9 (1.3–2.8)	<0·01	214 (55)	174 (45)	1.5 (1.1–1.9)	1.3 (0.9–1.8)	0.15
Symptom onset to diagnosis interval										
Short (0–4 wks.)	166 (84)	31 (16)	1 (Ref)	1 (Ref)		129 (65)	68 (35)	1 (Ref)	1 (Ref)	
Medium (5–11 wks.)	203 (81)	48 (19)	1.3 (0.8–2.1)	1.3 (0.8–2.3)	0.34	158 (63)	93 (37)	1.1 (0.8–1.6)	1.1 (0.7–1.7)	0.75
Long (≥12 wks.)	263 (74)	92 (26)	1.9 (1.2–2.9)	1.6 (0.9–2.6)	0.08	192 (54)	163 (46)	1.6 (1.1–2.3)	1.3 (0.9–2.0)	0.20
Total interval										
<91 days	240 (83)	49 (17)	1 (Ref)	1 (Ref)		193 (67)	96 (33)	1 (Ref)	1 (Ref)	
≥91 days	234 (75)	78 (25)	1.6 (1.1–2.4)	1.4 (0.9–2.3)	0.15	172 (55)	140 (45)	1.6 (1.2–2.3)	1.4 (0.9–2.1)	0.10

^a^Adjusted for gender, age group, deprivation, ethnicity, cancer site/type, marital status and education.

Participants with a patient interval of ≥4 weeks and those with a ‘symptom onset to oncology’ interval of ≥2 months were more likely to be clinically depressed (respective aORs and 95% CI: 1.7 (1.1–2.5) and 1.9 (1.3–2.8)). The point estimates of aORs were consistently above one (i.e. more likely to be clinically depressed) among those with more GP consultations, a longer ‘symptom onset to diagnosis’ interval and a longer total interval; however, there was insufficient evidence of group differences. Findings for the treatment interval are shown in Table S2.

### Diagnostic intervals and anxiety (Table 4)

Participants with ≥3 GP consultations prior to referral were more likely to be clinically anxious in crude and adjusted models (aOR: 1.6 95% CI 1.1–2.3). The point estimates of aORs for categories representing the longest intervals were consistently above one (i.e. higher rates of clinical anxiety) for those with a longer patient interval, ‘symptom onset to oncology’ interval, ‘symptom onset to diagnosis’ interval and total interval, although there was insufficient evidence of group differences.

Findings were similar for anxiety and depression when examined using the threshold for treatment (Supplementary Material Table S3), notably for longer ‘symptom onset to diagnosis’ intervals. Findings for the treatment interval are shown in Supplementary files Table S2.

## Discussion

We examined diagnostic/treatment intervals and their association with patient-reported outcomes in a large, well-characterised cohort of AYA. Consistent evidence existed (28/30 associations examined) that longer diagnostic/treatment intervals were associated with a higher risk of impaired HRQoL, clinical anxiety and clinical depression though there was often no evidence to support variation.

AYA experience longer cancer diagnostic intervals compared to children and adults [6, 16]. However, early diagnosis as a strategy to reduce cancer-related disease burden and improve outcomes for AYA receives minimal or no attention from global cancer control initiatives. This may in part reflect the lack of evidence about which outcomes may be improved. To our knowledge, our study is the first to report PROs associated with diagnostic/treatment intervals in a large cohort of AYA with cancer. Some prior evidence from adult patients suggests that longer diagnostic intervals are associated with poorer HRQoL [41], that rapid diagnostic pathways may be associated with reduced patient anxiety [42] and longer intervals are associated with higher HADS scores [42].

Our study represents novel empirical evidence of an association between longer time to diagnosis/treatment and poorer PROs among AYA patients. Acknowledging the relatively low incidence of cancer in this age group, our findings support further investigation of public health and system-level healthcare interventions aimed to expedite time to diagnosis and treatment. Cancer awareness in AYA is low [43], and although our data supports the need for AYA-targeted awareness campaigns to reduce the patient interval, the success of such interventions is difficult to measure. Additionally, there are likely to be psychological, social and circumstantial factors impacting the patient interval which are influential but may be out of reach of awareness campaigns. For example, cancer awareness campaigns in adolescents have improved symptom awareness but did not alter health-seeking behaviour. These included emotional factors, such as worrying about what the doctor might find, fear and embarrassment, social and circumstantial factors, such as arranging transport, being too busy and having other things to worry about [44, 45].

Expediting the time in primary care including referral to rapid diagnostic centres for symptomatic cancer patients receives considerable attention. The identification of positive predictive values (PPV) of ‘alarm symptoms’ in adults has provided useful decision support tools for GPs to support these initiatives. However, the data generated from PPV in AYA from single alert symptoms have been less enabling [46]. Further evaluation is needed of the PPV of multiple alarm and non-alarm symptoms together with consideration of other presenting features and medical history [41]. A GP may see only one or two AYA who go on to be diagnosed with cancer in their career, highlighting the difficulties of awareness campaigns for professionals as well as young people and the public. After referral into secondary care, the diagnostic experience of AYA is largely unknown, although recent reports suggest opportunities for improvement [15].

We previously reported subgroups of AYA at risk of longer times from symptom onset to diagnosis, time to diagnosis was not associated by age (younger teenagers versus young adults), socioeconomic status or ethnicity but was strongly associated with gender and cancer type [6]. In the BRIGHTLIGHT cohort those with leukaemia had shorter intervals to diagnosis, while longer times to diagnosis were observed in female AYAs and those with melanoma, lymphoma and bone tumours. These groups represent priority areas for further research. Also worthy of consideration is examining the association of diagnostic intervals on AYA aged 25–39 years, it is difficult to assess without examining whether the changing spectrum of incident cancers and increasing psychological maturity of young adults would generate the same results as we have observed in the 13–24 years old. Further research is needed to fully understand the challenges to identifying and diagnosing cancer in AYA, as well as innovation in diagnostic technologies for AYA.

Developing interventions to expedite AYA cancer diagnostic pathways is challenging. However, our results point to more achievable and amenable goals: the development and testing of interventions that mitigate the adverse impacts of prolonged diagnostic intervals may provide a more immediate effect to improve outcomes for young people with cancer. This will require further research with AYA and the professionals who care for them to identify what an intervention might look like, who is best placed to deliver it and when.

The societal gains of effective intervention are likely to be considerable given the years lived following a cancer diagnosis. The previously mentioned national research priority setting exercise for AYA placed ‘*What psychological support package improves psychological well-being, social functioning and mental health during and after treatment?* as the first research priority [18]. Our results suggest the psychological impact of cancer in AYA may begin in the pre-diagnostic period. Added to this, additional outcomes which are important to young people such as fertility, the financial impact of cancer, the impact of cancer on sexuality and identity may be impacted by diagnostic timeliness: these warrant further exploration.

We have investigated the relationship between diagnostic/treatment intervals and PROs among AYAs with cancer triangulating self-reported information, clinical data and health records. The BRIGHTLIGHT cohort is broadly similar to the incidence cases diagnosed during the same period of recruitment with some under-representation of brain and melanoma patients and over-representation of soft tissue sarcoma patients [9].

Despite this, our study has limitations. Our population is missing those who died within 6 months after diagnosis; this survivorship bias could have attenuated or augmented examined associations. Similarly, the BRIGHTLIGHT cohort has lower survival than the population not recruited but diagnosed during the same period, limiting generalisability [9]. Despite our multicentred study, the number in the cohort who died during follow-up was too small to look at the relationship between intervals and survival (data in the supplementary material to support future meta-analysis, Tables S4 and S5). BRIGHTLIGHT was designed to have a sufficient sample size for examining HRQoL as part of evaluating specialised AYA care, and not for the purposes of the present analysis. As the BRIGHTLIGHT protocol was not designed to examine diagnostic intervals, key dates such as the date of referrals from primary care to secondary care are missing, preventing us from examining the referral to secondary care interval intervals in days. However, the number of GP consultations is a good proxy indicator for the time in primary care [31]. We also acknowledge that our proxy indicator for the time in primary care was self-reported and therefore, may also be subject to recall bias. We were also unable to confidently examine the stage of disease at diagnosis due to incomplete data. Nevertheless, our study represents a significant contribution to begin to understand potential relationships between diagnostic/treatment intervals and outcomes in AYA given its uniquely large and representative sample.

## Conclusion

The association between time to cancer diagnosis and the outcomes we have examined is complex and challenging to study. In our study, we have identified AYA who take longer to be diagnosed with cancer are more likely to have impaired HRQoL and be clinically anxious or depressed. Diagnostic timeliness is multi-factorial with the complex interplay of patient, professional and healthcare system factors, not all of which are fully understood. We have identified multiple time intervals where further research to develop interventions to shorten the diagnostic timeliness could be targeted for AYA. We have shown a clear need for interventions that mitigate the adverse impacts of prolonged diagnostic intervals. Although implementation may be challenging, our findings shed light on an under-examined aspect of cancer control for AYA which has the potential to improve patient-reported outcomes.

## Supplementary information

Supplementary material

## Data Availability

Data are available upon reasonable request. Further details of the BRIGHTLIGHT programme of work are available through the study website (www.brightlightstudy.com). Data are available, except that held under licence with Public Health England or NHS Digital. We welcome collaboration, for general data sharing enquiries please contact LAF (lorna.fern@nhs.net).

## Code availability

Code is available on request.
