# Supplementary material for: Associations between diagnostic time intervals and health-related quality of life, clinical anxiety and depression in adolescents and young adults with cancer: cross-sectional analysis of the BRIGHTLIGHT cohort
Source: Br J Cancer. 2022 Feb 22;126(12):1725–34. doi: 10.1038/s41416-022-01698-6 (PMC9174449; doi:10.1038/s41416-022-01698-6)
Supplement: Supplementary file 1 — Supplementary material [file 41416_2022_1698_MOESM1_ESM.docx]

**Supplementary Material**

**Table S1 – Demographic and clinical characteristics of the sample**

|  | **n (%)** |
| --- | --- |
| **Gender** |  |
| Male | 453 (55) |
| Female | 377 (45) |
|  |  |
| **Age group** |  |
| 12-15 years | 115 (14) |
| 16-18 years | 187 (23) |
| 19-24 years | 528 (64) |
|  |  |
| **Ethnicity** |  |
| White | 730 (88) |
| Not White | 100 (12) |
|  |  |
| **Index of multiple deprivation quintile** |  |
| 1 (least deprived) | 184 (23) |
| 2 | 136 (17) |
| 3 | 156 (19) |
| 4 | 182 (22) |
| 5 (most deprived) | 158 (19) |
|  |  |
| **Employment status** |  |
| In education | 274 (33) |
| Working full or part time | 257 (31) |
| Other work (apprentice/intern/voluntary) | 17 (2) |
| Not seeking work | 125 (15) |
| Unemployed | 31 (4) |
| Long-term sick | 126 (15) |
|  |  |
| **Marital status** |  |
| Married / civil partnership | 119 (14) |
| Cohabiting / single / divorced | 709 (86) |
|  |  |
| **Cancer type** |  |
| Lymphoma | 266 (32) |
| Germ cell | 156 (19) |
| Leukaemia | 105 (13) |
| Carcinomas | 100 (12) |
| Bone | 79 (10) |
| Soft tissue sarcoma | 50 (6) |
| Central nervous system | 33 (4) |
| Melanoma | 31 (4) |
| Unclassified or unspecified | 10 (1) |
|  |  |
| **Treatment type** |  |
| Chemotherapy only | 271 (33) |
| Surgery and chemotherapy | 181 (22) |
| Surgery only | 117 (14) |
| Chemotherapy and radiotherapy | 106 (13) |
| Surgery, radiotherapy and chemotherapy | 77 (9) |
| Surgery and radiotherapy | 36 (4) |
| Radiotherapy only | 14 (2) |
| Transplant only | 19 (2) |
| Other | 9 (1) |
|  |  |
| **Category of AYA specialist care** |  |
| No AYA care | 277 (35) |
| Some AYA care | 312 (40) |
| All AYA care | 193 (25) |

a Percentages not equal to 830 due to missing data.

***Table S2 – Adjusted regression models exploring associations between the treatment interval and patient-reported outcomes***

|  | **QoL** | | **Clinical depression** | | **Clinical anxiety** | | **Moderate / severe depression** | | **Moderate / severe anxiety** | |
| --- | --- | --- | --- | --- | --- | --- | --- | --- | --- | --- |
|  | **High risk of impaired HRQoL**  n (%) | **Adjusted ^a^ odds ratio**  **(95% CI)**  **p value** | **Clinically depressed**  n (%) | **Adjusted ^b^ odds ratio**  **(95% CI)**  **p value** | **Clinically anxious**  n (%) | **Adjusted ^b^ odds ratio**  **(95% CI)**  **p value** | **Moderately/severely depressed**  n (%) | **Adjusted ^b^ odds ratio**  **(95% CI)**  **p value** | **Moderately/severely anxious** | **Adjusted ^b^ odds ratio**  **(95% CI)**  **p value** |
| **Treatment interval** |  |  |  |  |  |  |  |  |  |  |
| ≤31 days | 231 (57) | 1 (Ref) | 82 (20) | 140 (35) | 1 (Ref) | 1 (Ref) | 22 (5) | 1 (Ref) | 76 (18) | 1 (Ref) |
| >31 days | 89 (61) | 1⋅1  (0⋅6-1⋅8)  0⋅82 | 33 (23) | 73 (50) | 1⋅5  (0⋅9-2⋅3)  0⋅11 | 0⋅8  (0⋅5-1⋅4)  0⋅43 | 12 (8) | 1⋅2  (0⋅5-2⋅8)  0⋅70 | 36 (23) | 1⋅3  (0⋅6-1⋅8)  0⋅90 |

a Adjusted for gender, age group, deprivation, ethnicity, cancer site/type, marital status, education, treatment type and level of specialist care.

b Adjusted for gender, age group, deprivation, ethnicity, cancer site/type, marital status, and education

***Table S3 - Crude and adjusted logistic regression models exploring associations between diagnostic and treatment intervals and depression and anxiety at the threshold for treatment***

|  | **Depression** | | | | | **Anxiety** | | | | |
| --- | --- | --- | --- | --- | --- | --- | --- | --- | --- | --- |
|  | **Not clinically depressed**  n (%) | **Depression at treatment threshold**  n (%) | **Crude hazard ratio**  (95% CI) | **Adjusted ^a^ OR**  (95% CI) | **Adjusted p value** | **Not clinically anxious**  n (%) | **Anxiety at treatment threshold**  n (%) | **Crude OR** (95% CI) | **Adjusted ^a^ OR**  (95% CI) | **Adjusted p value** |
| **Patient interval** |  |  |  |  |  |  |  |  |  |  |
| <4 weeks | 515 (95) | 29 (5) | 1 (Ref) | 1 (Ref) |  | 439 (81) | 105 (19) | 1 (Ref) | 1 (Ref) |  |
| ≥4 weeks | 184 (90) | 20 (10) | 1⋅9  (1⋅1-3⋅5) | 2⋅0  (1⋅1-3⋅8) | 0⋅03 | 154 (75) | 50 (25) | 1⋅4  (0⋅9-2⋅0) | 1⋅1  (0⋅7-1⋅7) | 0⋅6 |
| **GP consultations (number)** |  |  |  |  |  |  |  |  |  |  |
| 1-2 | 433 (94) | 26 (6) | 1 (Ref) | 1 (Ref) |  | 380 (83) | 79 (17) | 1 (Ref) | 1 (Ref) |  |
| ≥3 | 227 (94) | 15 (6) | 1⋅1  (0⋅6-2⋅1) | 0⋅7  (0⋅3-1⋅5) | 0⋅34 | 179 (74) | 63 (26) | 1⋅7  (1⋅1-2⋅5) | 1⋅4  (0⋅9-2⋅1) | 0⋅13 |
| **Symptom onset to oncology** |  |  |  |  |  |  |  |  |  |  |
| <2 months | 418 (95) | 21 (5) | 1 (Ref) | 1 (Ref) |  | 371 (85) | 68 (15) | 1 (Ref) | 1 (Ref) |  |
| ≥2 months | 354 (91) | 34 (9) | 1⋅9  (1⋅1-3⋅4) | 1⋅6  (0⋅9-3⋅0) | 0⋅12 | 284 (73) | 104 (27) | 2⋅0  (1⋅4-2⋅8) | 1.8  (1⋅2-2⋅7) | <0⋅01 |
| **Symptom onset to diagnosis interval** |  |  |  |  |  |  |  |  |  |  |
| Short (0-4 wks.) | 192 (97) | 5 (3) | 1 (Ref) | 1 (Ref) |  | 167 (85) | 30 (15) | 1 (Ref) | 1 (Ref) |  |
| Medium  (5-11 wks.) | 231 (92) | 20 (8) | 3⋅3  (1⋅2-9⋅0) | 3⋅2  (1⋅1-9⋅1) | 0⋅03 | 203 (81) | 48 (19 | 1⋅3  (0⋅8-2⋅2) | 1⋅5  (0⋅9-2⋅7) | 0⋅16 |
| Long (≥12 wks.) | 326 (92) | 29 (8) | 3⋅4  (1⋅3-9⋅0) | 2⋅7  (1⋅0-7⋅4) | 0⋅06 | 264 (75) | 91 (25) | 1⋅9  (0⋅8-2⋅2) | 1.7  (1⋅0-2⋅9) | 0⋅05 |
| **Total interval** |  |  |  |  |  |  |  |  |  |  |
| <91 days | 278 (96) | 11 (4) | 1 (Ref) | 1 (Ref) |  | 243 (84) | 46 (16) | 1 (Ref) | 1 (Ref) |  |
| ≥91 days | 286 (92) | 26 (6) | 2⋅3  (1⋅1-4⋅7) | 2⋅1  (0⋅9-4⋅9) | 0⋅08 | 236 (76) | 76 (24) | 1⋅7  (1⋅1-2⋅6) | 1⋅5  (0⋅9-2⋅4) | 0⋅10 |

a Adjusted for gender, age group, deprivation, ethnicity, cancer site/type, marital status and education

**Diagnostic and treatment intervals and survival**

*Methods*

Survival was calculated from date-of-diagnosis to date-of-death or date last known to be alive up to 29^th^ October 2018, obtained from NHS digital. To explore if there were differences in survival by diagnostic and treatment intervals, we present a time-to-event analysis for each interval variable. We report crude and adjusted cox model estimates (adjusting for gender, age group, deprivation, ethnicity, cancer site/type, marital status, and employment status) with 95% CI. The proportional hazards assumption was tested and confirmed using the ‘estat phtest’ command in STATA.

*Results*

**Table S4 – Survival at 1 and 3 years post-diagnosis**

|  | n (%) |
| --- | --- |
| Alive at 1 year | 822 (99) |
| Alive at 3 years | 751 (90) |

**Table S5 - *Crude and adjusted regression models exploring associations between survival and diagnostic and treatment intervals***

|  | **Alive at 3 years**  n (%) | **Dead at 3 years**  n (%) | **Crude hazard ratio** (95% CI) | **Adjusted ^a^ hazard ratio**  (95% CI) | **Adjusted p value** |
| --- | --- | --- | --- | --- | --- |
| **Patient interval** |  |  |  |  |  |
| <4 weeks | 494 (91) | 50 (9) | 1 (Ref) | 1 (Ref) |  |
| ≥4 weeks | 183 (90) | 21 (10) | 1⋅8 (0⋅8-1⋅9) | 1.2  (0⋅7-1⋅9) | 0⋅51 |
| **GP consultations** (number) |  |  |  |  |  |
| 1-2 | 331 (93) | 26 (7) | 1 (Ref) | 1 (Ref) |  |
| ≥3 | 210 (87) | 32 (13) | 1⋅4 (0⋅9-2⋅6) | 1⋅5  (0⋅9-2⋅4) | 0⋅08 |
| **Symptom onset to oncology** |  |  |  |  |  |
| <2 months | 405 (92) | 34 (8) | 1 (Ref) | 1 (Ref) |  |
| ≥2 months | 343 (88) | 45 (12) | 1.3  (0⋅9-1⋅9) | 1⋅0  (0⋅7-1⋅5) | 0⋅85 |
| **Symptom onset to diagnosis interval** |  |  |  |  |  |
| Short (0-4 wks.) | 175 (89) | 22 (11) | 1 (Ref) | 1 (Ref) |  |
| Medium (5-11 wks.) | 236 (94) | 15 (6) | 0⋅5  (0⋅3-0⋅9) | 0⋅5  (0⋅3-0⋅9) | 0⋅03 |
| Long (≥12 wks.) | 315 (89) | 40 (11) | 1⋅0  (0⋅6-1⋅6) | 0⋅8  (0⋅5-1⋅3) | 0⋅47 |
| **Total interval** |  |  |  |  |  |
| <91 days | 268 (93) | 21 (7) | 1 (Ref) | 1 (Ref) |  |
| ≥91 days | 278 (89) | 34 (11) | 1⋅4  (0⋅9-2⋅3) | 1⋅2  (0⋅7-2⋅0) | 0⋅46 |

a Adjusted for gender, age group, deprivation, ethnicity, cancer site/type, marital status, and education
